# Characterization of *Salmonella* Phage LPST153 That Effectively Targets Most Prevalent *Salmonella* Serovars

**DOI:** 10.3390/microorganisms8071089

**Published:** 2020-07-21

**Authors:** Md. Sharifull Islam, Yang Hu, Md. Furkanur Rahaman Mizan, Ting Yan, Ishatur Nime, Yang Zhou, Jinquan Li

**Affiliations:** 1 College of Fisheries, Huazhong Agricultural University, Wuhan 430070, China; smbgb101287@yahoo.com; 2 State Key Laboratory of Agricultural Microbiology, Huazhong Agricultural University, Wuhan 430070, China; lijinquan2007@gmail.com; 3 Key Laboratory of Environment Correlative Dietology, College of Food Science and Technology, Huazhong Agricultural University, Wuhan 430070, China; yantingau@163.com (T.Y.); smbgb101287@gmail.com (I.N.); 4 Division of Microbiology, Brewing and Biotechnology, School of Biosciences, University of Nottingham, Sutton Bonington Campus, Loughborough, Leicestershire LE12 5RD, UK; stxyh26@exmail.nottingham.ac.uk; 5 Department of Food Science and Technology, Chung-Ang University, Anseong, Gyunggido 456-756, Korea; rahaman4162@cau.ac.kr; 6 Laboratory of Bacterial Pathogenesis and Immunology, The Rockefeller University, New York, NY 10065-6399, USA

**Keywords:** *Salmonella*, phage, LPST153, halo, food, biofilm

## Abstract

Foodborne diseases represent a major risk to public health worldwide. In this study, LPST153, a novel *Salmonella* lytic phage with halo (indicative of potential depolymerase activity) was isolated by employing *Salmonella enterica* serovar Typhimurium ATCC 13311 as the host and had excellent lytic potential against *Salmonella*. LPST153 is effectively able to lyse most prevalent tested serotypes of *Salmonella*, including *S.* Typhimurium, *S.* Enteritidis, *S.* Pullorum and *S.* Gallinarum. Morphological analysis revealed that phage LPST153 belongs to *Podoviridae* family and *Caudovirales* order and could completely prevent host bacterial growth within 9 h at multiplicity of infection (MOI) of 0.1, 1, 10 and 100. LPST153 had a latent period of 10 min and a burst size of 113 ± 8 PFU/cell. Characterization of the phage LPST153 revealed that it would be active and stable in some harsh environments or in different conditions of food processing and storage. After genome sequencing and phylogenetic analysis, it is confirmed that LPST153 is a new member of the *Teseptimavirus* genus of *Autographivirinae* subfamily. Further application experiments showed that this phage has potential in controlling *Salmonella* in milk and sausage. LPST153 was also able to inhibit the formation of biofilms and it had the ability to reduce and kill bacteria from inside, including existing biofilms. Therefore, the phage LPST153 could be used as a potential antibacterial agent for *Salmonella* control in the food industry.

## 1. Introduction

*Salmonella* is a member of the *Enterobacteriaceae* family, a globally important foodborne pathogen which has caused diseases known as salmonellosis. Nontyphoidal *Salmonella* has been associated with many food-borne diseases across the world. The most prevalent *Salmonella* serovars are including *S.* Typhimurium, *S.* Enteritidis, *S*. Pullorum and *S.* Gallinarum, among them, *S.* Typhimurium and *S.* Enteritidis have been described as worldwide spread [1,2,3]. Some of these prevalent serovars could lead to serious zoonotic diseases [4]. While *S.* Pullorum and *S.* Gallinarum are connected with diseases in poultry and they were reported to cause financial sufferers because of the replacement of infected flocks and associated treatment costs to poultry farmers [5]. Nontyphoidal *Salmonella* spp. is a human foodborne pathogen which was usually responsible for 93 million infections annually worldwide [6]. It has been reported to cause at least 93.8 million relative diseases, 155,000 deaths and about 40,000 patients suffering with salmonellosis annually in the United States [7]. So, *Salmonella* is considered as one of the major foodborne pathogens [7]. It has been claimed that most of the *Salmonella* infections are caused by consumption of *Salmonella* contaminated foods, including poultry, eggs, dairy products, fresh fruits and vegetables [8]. Inappropriate use of antibiotics in poultry and other farm animals resulted in antibiotic-resistant *Salmonella* isolated becoming more and more frequent from clinical samples. Some *Salmonella* spp. has even risen to multidrug resistance (MDR), which could pose serious threats to public health [9].

It has been known that bacteria in biofilm are highly related with its resistance to antimicrobials and multidrug resistance. Biofilms are defined as highly organized multicellular communities of bacteria enclosed in extracellular polymeric substances (EPS) which can protect the encased microorganisms from host’s immune system attacking [10,11]. Most of the pathogenic bacteria are able to form biofilms on a variety of materials, such as, plastic, metal, glass, wood, and many other food stuffs [12,13].

Phages are viruses which can target and kill bacteria, so that phages are considered as one kind of alternative for antibiotics or chemicals [14]. The first reported application of therapeutic phages dates can be traced back to the early 20th century [15] while phages were used to control a variety of diseases including diarrhea, cholera, dysentery and salmonellosis [16]. Until now, phage therapy has been used for inactivation and controlling of food-borne pathogens; such as *Salmonella*, *Listeria*, *Campylobacter* and *Escherichia coli* O157:H7 in diverse foods [17,18,19,20,21,22,23,24,25,26,27,28]. In addition, commercially recognized phage products, such as Armament, Salmonelex, and SalmoFresh have been used for inactivating and controlling *Salmonella* in food products [29,30]. Since bacteria and their phages have constantly coevolved for billions of years, bacteria could rise some mechanisms in defending against phages under some circumstances [31,32]. In order to counteract these defense mechanisms, it is required to continuously provide new promising phages with broad range and high lytic capacity for practical application. Current interests in biocontrol phages predominately involve the disinfection of food product surfaces and production equipment’s during food processing, thus preventing cross-contamination of the final product [33].

In this study, LPST153, a novel *Salmonella* lytic phage with halo was isolated and has lytic potential against *Salmonella.* Morphology, pH stability and thermal stability of LPST153 were evaluated. As a biological control agent, the potential efficiency of LPST153 against *Salmonella* in food matrices and biofilm was also tested.

## 2. Materials and Methods

### 2.1. Bacterial Strains and Culture Conditions

For phages isolation, *S*. Typhimurium ATCC 13311 was employed as the host strain. A total of 65 diverse bacterial strains were used for the phage host range experiments (Table 1), which consist of 41 *Salmonella* strains including 13 distinct serovars and 24 non-*Salmonella* strains. All the strains were cultivated by the streak plate method on tryptic soy agar (TSA; Difco, BD, Franklin Lakes, NJ, USA) then overnight incubation at 37 °C.

### 2.2. Enrichment, Isolation, Purification, and Preparation of Phages

A total of 12 water samples were collected from different parts of east and west lake, Wuhan, China. Samples were screened and 12 putative different phages including phage LPST153 were isolated and *S.* Typhimurium ATCC 13311 were used as hosts in this study. For enrichment, applied phages isolation and purification, modified methods were the same as the previously published article [34]. In brief, 10 mL of a 0.22-µm-filtered water sample, 10 mL fresh *Salmonella* cultures (about 8 log_10_ CFU/mL), and 40 mL tryptone soya broth (TSB) medium were mixed together. Then, the mixture was incubated at 37 °C for 24 h and agitated at 160 rpm/min. Following incubation, the cultures were centrifuged for 15 min at 8000× *g* and the supernatants were filtered through 0.22 µm filters (Millipore, Cork, Ireland). The presence of phages in the supernatant were confirmed by spot assay. Phages were isolated and purified by using standard plaque assays after resuspension in TSB, in which *S.* Typhimurium ATCC 13311 was employed as host. The phage purification process was repeated at least 4 times. The purified phages were stored in TSB and with 20% glycerol (v/v) applied at −80 °C for further use.

### 2.3. Morphological Observation of Phage LPST153

Ten microliter lysate with high titers (>10^10^ PFU/mL) of purified phage was fixed onto a copper grid and negatively stained with 2% phosphotungstic acid (PTA) [35]. Several series of phage photographs were captured under a Philips CM12 transmission electron microscope (Hitachi H-7000FA, Tokyo, Japan), in Wuhan Institute of Virology (China Academy of Sciences, Wuhan, China).

### 2.4. Spot Test

In total, 65 diverse bacterial strains were used to examine the phage lytic range, which consisted of one group of 41 *Salmonella* strains and a group of 24 non-*Salmonella* strains; there were 13 different serovars in *Salmonella* group (Table 1). The ability of phages to infect bacteria with different serovars was determined by spot test. In brief, the method can be described as follows: 5 μL lysates from each phage were spotted onto bacteria lawns which were separately poured with 65 bacterial strains, in which the strains were propagated on TSA plates. Four microliter 0.7% soft agar overlay was applied to enable clear spots/plaques development. The lytic range activities of all bacterial strains were determined at 37 °C with 24 h incubation. After the incubation, clear spots/plaques on any bacterial lawns were recorded as corresponding phage sensitive.

### 2.5. Determination of Host Range by Efficiency of Plating (EOP)

To determine the host range, efficiency of plating (EOP) has been considered as a described method in previous reports with limited modifications [36,37]. Series dilution has been applied on phage lysate, in which four levels of dilution were applied on all the testing stains. All the processes have been repeated for at least three times as both biological control and technique control. One hundred microliter of each testing fresh culture of bacteria (approximately 10^8^ CFU/mL) was applied in double layer plate assays together with 100 μL of diluted phage lysate. The four phage lysates were diluted to between 10^−6^ and 10^−9^ multiples of the phage stock. The plates were incubated overnight at 37 °C and the number of plaques was counted by the next day, so that plaque forming units (PFU) can be calculated. Finally, the EOP was calculated (EOP = average PFU on test bacteria/average PFU on host bacteria). The EOP assessment was categorized as EOP 0.5 to 1.0, high efficiency; EOP 0.2 to <0.5, moderate efficiency; 0.001 to <0.2, low efficiency; and <0.001, inefficient [36,37].

### 2.6. Lytic Activity

The lytic activity experiment was supposed for the evaluating efficiency of phages’ virulence. The optical density (OD_600nm_) was measured in the 96-well microtiter plate every hour at various multiplicity of infection (MOI; ratio of phage titers to bacterial counts measured) from 0.1, 1, 10 and 100. The hypothesis of lytic activity experiments was described in previously published paper method [36]. In brief, the test groups which contain 100 μL of fresh overnight cultures of *Salmonella* (10^7^ CFU/mL) were mixed with 100 μL diluted phage lysate (10^6^–10^9^ PFU/mL) in the wells of 96 well-microtiter plate. Each dilute level has been covered in this process. The control groups remain the same as test groups in volume, which consist of fresh overnight cultures of *Salmonella* (10^7^ CFU/mL), but phage suspension was replaced by plain TSB medium. The test and control groups were both incubated in exactly same conditions (shaking at 160 rpm under 37 °C). The optical density (OD_600nm_) of the mixture was measured with a microplate reader (Infinite M200 Pro, Tecan, Männedorf, Switzerland) at 37 °C, every 1 h intervals.

### 2.7. One-Step Growth Curve

One-step growth curve experiments have been accomplished to conclude the latent period and burst size according to previously introduced methods [38,39], with modifications on time points. Firstly, *S.* Typhimurium ATCC 13311 was allowed to grow to mid-log phase and, then, 1 mL of bacterial culture (1.5 × 10^7^ CFU/mL) which has been combined with 1 mL of phage lysate (1.5 × 10^4^ PFU/mL) was added to achieve a multiplicity of infection (MOI) of 0.001. The mixture was incubated for 10 min at 37 °C and was subsequently centrifuged at 7000× *g* for 2 min. Afterwards, the supernatant was discarded and the pellets were washed twice with TSB. Then the pellets were suspended into 10 mL of TSB broth and the broth was incubated at 37 °C. After incubation, the broth was aliquoted to 200 μL. Every 200 μL broth was collected at every 10 min interval in total 180 min. Finally, the phage titers were calculated by the double layer agar plate method [40] to gain one-step growth curve. The latent period was defined as the time interval between absorption and the beginning of the first burst. The burst size was summarized as the ratio of the final number of phage particles and the initial number of host bacteria at the beginning of the experiment [38,39].

### 2.8. pH and Thermal Tolerance of the Phage LPST153

To determine pH effecting on the stability of phage LPST153, phage lysates (3.5 × 10^8^ PFU/mL) and sterile TSB were added to test tubes. Values of pH were ranging from 2 to 13 adjusted with NaOH or HCl. Then, the tubes were incubated at 37 °C for 60 min. Thereafter, the samples were diluted and phage titers were recovered by using *S.* Typhimurium ATCC 13311 as a host in double-layer agar method [40]. The samples of the phage LPST153 lysates (1.5 × 10^8^ PFU/mL) were incubated at 30, 40, 50, 60, 70 and 80 °C for investigating the thermal stability. After 30 or 60 min of incubation, aliquots were collected for determining phage titers by using double-layer agar method.

### 2.9. Phage Genome Sequencing, Genome Annotation and Comparison

Phage genomic DNA was isolated and purified as previously described by Yan et al. [41]. Briefly, 1 mL lysate with a high titer (>10^10^ PFU/mL) of purified phage was used for DNA isolation. To degrade the bacterial nucleic acid, 20 μL of deoxyribonuclease DNase I (1 mg/mL) and 20 μL ribonuclease RNase A (10 mg/mL) were added to the phage suspension and vortexed for 2 min, then, incubated at 37 °C for 40 min. Following incubation, 20 μL of 2M ZnCl_2_ was added and the mixture was incubated at 37 °C for another 7 min. After this incubation, the mixture was centrifuged at 10,000 rpm for 1 min and discarded the supernatant. The pellets were resuspended in 500 µL of phage buffer. The phage suspension was used for DNA by phenol:chloroform protocol. The DNA library was prepared by using Illumina TruSeq DNA Nano Library Prep Kit, according to the manufacturer’s instructions. The genome was sequenced by employing the Illumina HiSeq 4000 sequencing platform with a paired-end read length of 2 × 150 bp, producing assembly sequences correction, respectively, and then genome was assembled by MicrobeTrakr plus (v0.9.1) software. Open reading frames (ORFs) of LPST153 were also predicted by using MicrobeTrakr plus (v0.9.1) software. Annotation of predicted ORFs was accomplished by BLASTP [42] and CD searching against (NCBI) nonredundant database, the Conserved Domain Database (CDD), Pfam, SMART, COG, and InterProScan5 [43]. The complete genome sequence was deposited in GenBank under the accession number MK907285.

Multiple sequence alignment of the terminase large subunit and capsid protein amino acid sequences were performed by a ClustalW algorithm and related phylogenetic tree was generated using the MEGA6 program via the neighbor-joining method with 1000 bootstrap replicates [44] and optimized by online website tool ITOL (https://itol.embl.de/).

### 2.10. Biological Control of Salmonella in Foods Using Phage LPST153

Pasteurized milk was purchased from a local supermarket in Wuhan, China. *Salmonella* biocontrol experiments employed phage LPST153 which was conducted at 4 °C (refrigerator temperature) and 25 °C (room temperature) [45]. *S.* Typhimurium ATCC 13311 was mixed with raw milk to a final count of 3.8 log_10_ CFU/mL. Then, phage LPST153 was added and MOI was controlled at either 1000 or 10,000. Aliquots were collected at 0, 1, 3, 6, and 9 h incubation and recoverable bacteria were quantified by direct plating.

Packed raw beef sausage (Smoky and Spicy, China Xiangtai Food co., Ltd., Chongqing, China.) was purchased from the same supermarket then sliced aseptically in the laboratory. Raw sausages were cut into cubes (1 cm × 1 cm square and 0.5 cm thick) by using a sterile sharp knife offered by laboratory sterile station. Then, processed sausage cubes were placed in the center of the sterile petri-dishes and 10 μL of *S*. Typhimurium ATCC 13311 with final concentration of 4 log_10_ CFU/cm^2^ was inoculated. Ten microliter phage LPST153 lysate (with a final concentration of 7 log_10_ and 8 log_10_ PFU/mL) spotted on the sample surface with the MOI controlled at 1000 or 10,000. Petri-dishes which contained the samples were incubated at 4 °C or 25 °C for 9 h with cap covered. At the time point 0, 1, 3, 6, and 9 h, samples were transferred to 2 mL Eppendorf tubes separately. Thereafter, 1 mL of PBS buffer was added to each sample which has been transferred into Eppendorf tubes with sterile operations. The sausage samples were homogenized with sterile bars stirring and then vortexed for 5 min. The numbers of bacteria recovered from the control and the experimental group were determined by direct spread plate methods.

### 2.11. Biofilm Assay in 96-Well Microplate

Two strategies have been identified by using phages for preventing biofilms forming, on one hand, blocking or reducing biofilm development from forming and on another hand, reduction of an existing biofilm [46,47]. Colorimetric method was carried out to reveal phage LPST153 effecting on inhibiting biofilm formation and reducing already formed biofilm of *S.* Typhimurium ATCC 13311 [48]. On one of the 96-well microplates, *S.* Typhimurium ATCC 13311 was inoculated into fresh LB at a final concentration of 4 log_10_ CFU/mL in every well. This 96-well microplate was incubated at 30 °C (optimal temperature for biofilm formation) [49] for 72 h under static condition for bacteria attaching on the wall of well and further forming biofilms. For inhibiting biofilm formation, phage lysate was added into the bacterial mixtures at a final titer of 7 log_10_ and 8 log10 PFU/mL after 72 h 30 °C incubation, so that already formed biofilms were treated with phage LPST153 at a final titer of 7 log_10_ and 8 log_10_ PFU/mL. In the control group, phosphate-buffered saline (PBS) was used instead of phage lysate. Samples were still incubated at 30 °C for another 12 h. After phage treatment, each well was rinsed with PBS for 5 times and allowed to dry in the laminar air-flow cabinet. After rinse with PBS, 98% methanol was applied and kept for 10 min. The methanol was then removed by pipetting, and plates were allowed to dry in laminar air-flow cabinet again. Then 200 μL of 1% crystal violet solution was added to the each well and kept for 45 min. Finally, it was eluted with 200 μL of 33% acetic acid. The OD values of eluted samples were measured by a spectrometer (Infinite M200 Pro, Tecan, Männedorf, Switzerland) at a wavelength of 600 nm.

### 2.12. Statistical Analysis

Statistical analysis was conducted by using Prism 6.01 for Windows (GraphPad software, San Diego, CA, USA). Multivariate comparisons were done by using nonparametric one-way analysis of variance (ANOVA) and Bonferroni’s multiple-comparison posttest has been applied as well. Mean values of each group’s data are presented with standard deviations. Statistical significance was considered at significance level only if and when *p* < 0.05.

## 3. Results

### 3.1. Morphology of LPST153 with Halo Zone

**Figure 1:**
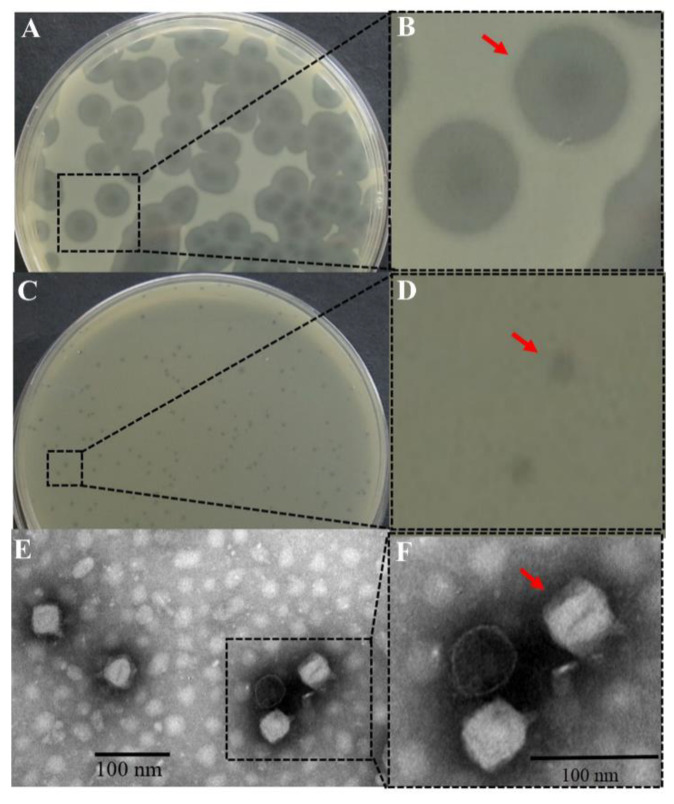
Morphology of phage. (**A**) Plaque morphologies of phage LPST153, the incubation time was 24 h, (**B**) Halo zone of phage LPST153, (**C**) Plaques morphologies of phage LPST89; the incubation time was 24 h, (**D**) No halo zone of phage LPST89 was observed, and (**E**,**F**) Representative TEM image of phage LPST153.

A total of 12 putative different phages encompassing phage LPST153 were isolated from water sample while *S.* Typhimurium ATCC 13311 was employed as host strain in laboratory practices. The plaque morphologies of phages LPST153 and LPST89 are shown in Figure 1A,C. It was confirmed that each single phage LPST153 could produce one relatively large and clear plaque (diameter, 3.2 ± 0.2 mm) with a halo zone (diameter, 8.5 ± 0.4 mm) within 24 h. However, LPST89 created smaller plaques than LPST153 (0.5 to 1.0 mm in diameter) which remained approximately the same size after 24 h of incubation. It is believed that phenotype of halo zone is an indication of the ability to depolymerize exopolysaccharide and biofilm [50]. LPST153 with halo zone (Figure 1B) was chosen for further study. Transmission electron microscopy (TEM) revealed that LPST153 consisted of an icosahedral head which was 51.5 ± 4.5 nm in diameter (*n* = 6) and a short tail which was 7.5 ± 2.4 nm in length (*n* = 6). The morphology was thus similar to a typical member of *Podoviridae* family (Figure 1E,F).

### 3.2. Host Range of LPST153

Analysis of the lytic range of phage LPST153 revealed that this phage lysed all 30 (100%) tested strains which come from 4 most prevalent *Salmonella* serovars (*S.* Typhimurium, *S.* Enteritidis, *S.* Pullorum and *S.* Gallinarum) in which some strains were drug-resistant (Table 1). Four types of tested *Salmonella* have risen their resistance to LPST153, including *S.* Dublin LSD2, *S*. Anatum ATCC 9270, *S.* Kentucky LSX24, and *S.* Newport E20002725. However, LPST153 was incapable to lyse *E. coli* or other tested non-*Salmonella* bacteria (Table 1). Moreover, host range results of LPST153 showed that it could lyse *S.* Typhimurium (*n* = 6), *S.* Enteritidis (*n* = 4), *S.* Pullorum (*n* = 5) and *S.* Gallinarum (*n* = 5), the EOP values (0.1 to 1.0). The EOP values were from 0.001 to less than 0.2 or inefficient for other bacterial serovars tested in this study (Table 1).

### 3.3. Characteristics of LPST153

**Figure 2:**
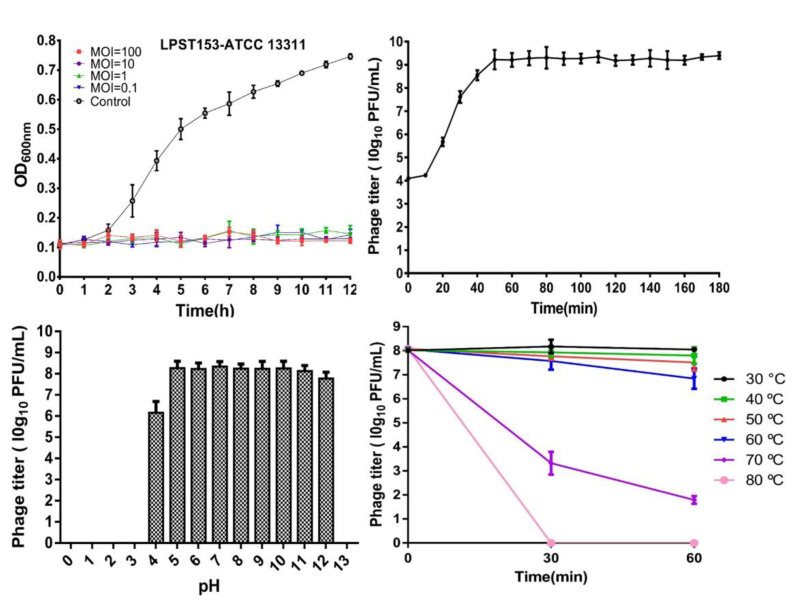
Characteristics of LPST153. (**A**) Lytic ability of phage LPST153 to lyse *S. enterica* serovar Typhimurium ATCC 13311 in TSB medium at different MOIs of 100, 10, 1 and 0.1 at 37 °C, (**B**) One step growth curve of LPST153, (**C**) pH stability, LPST153 exhibited pH stability range from 4 to 12, and (**D**) Temperature stability. LPST153 exhibited stable at 30 °C to 60 °C. Data were shown as mean with standard deviation of three determinations of each point.

The lytic activity of phage LPST153 was investigated by infecting *S*. Typhimurium ATCC 13311 in liquid cultures (Figure 2A). As shown in Figure 2A, phage LPST153 could constantly inhibit the growth of *S.* Typhimurium ATCC 13311 with less counts at MOI ratios of 0.1, 1, 10 and 100 in 12 h (*p* < 0.05). The one-step growth curve of LPST153 was showed in Figure 2B. The latent period of LPST153 was observed within 10 min and the burst size was approximately 113 ± 8 PFU/cell. For confirming application of LPST153 as a biocontrol agent to inhibit pathogenic bacteria, its viable stability would be needed to be confirmed under various stress conditions, such as pH and temperature. The pH stability test of the LPST153 showed that it was highly stable with environmental pH flowing from 4 to 12. However, this phage was completely abolished under strong acid or strong alkali (pH < 4 or pH > 12) (Figure 2C). There was no significant loss of LPST153 phage count between 30 °C and 60 °C, while it is the optimum temperature for phage stability from 30 °C to 50 °C. However, the phage count was reduced by approximately 75% at 70 °C, indicating that phage LPST153 has moderate heat resistance (Figure 2D).

### 3.4. Genomic Characterization of LPST153

**Figure 3:**
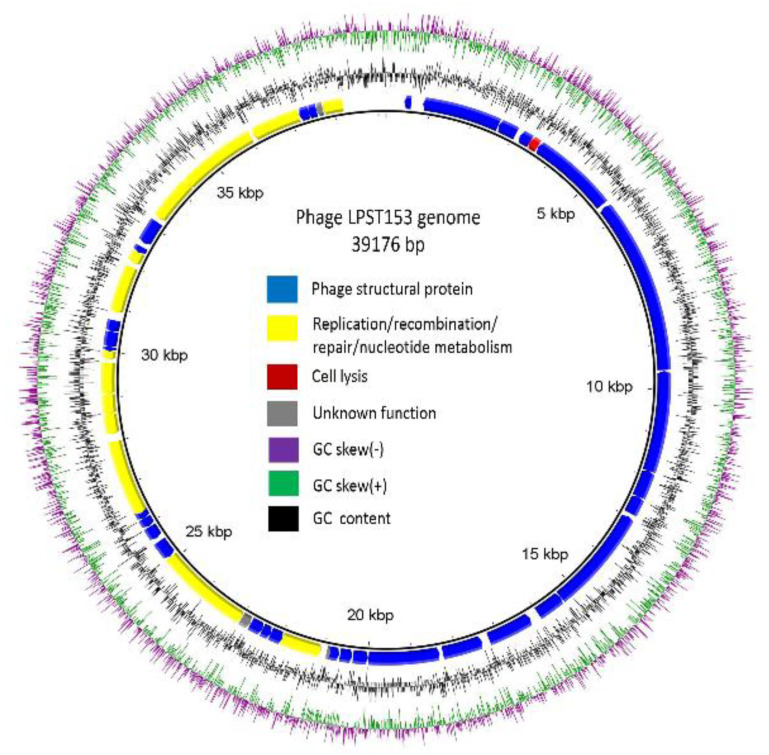
Genome map of phage LPST153. Genome arrangements were divided into four circles: The inner circle indicates the full length of the genome; the second circle indicates the gene coding regions by strand and the clockwise arrow and the counterclockwise arrow denoted the forward reading frame and the reverse reading frame, respectively. The color of each gene refers to the functional category: phage structure (blue), replication/recombination/repair/nucleotide metabolism (yellow), cell lysis (red) and hypothetical proteins (grey). The third circle with a black line indicates the GC content. The outer circle indicates GC skew of G-C/G+C as green and purple: green denotes the values of GC skew were greater than 0 and purple denotes the values were less than 0.

The genome of LPST153 is double-stranded DNA of 39,176 bp, with 49.1% GC content (Figure 3). The putative open reading frames (ORFs) were predicted by using RAST server [51] and it showed that LPST153 genome contains 48 ORFs (Table S1). Forty-five of the forty-eight ORFs were predicted to reveal identified functions, which include packaging module and structure, the replication/transcription module and the host lysis module. As shown in Figure 3, the DNA packaging module was composed of two DNA packaging ORFs (DNA packaging protein A and DNA packaging protein B). The main structural genes are: capsid proteins A, B, C and D (gp13, gp14, gp15 and gp16), capsid and scaffold proteins (gp9 and gp10A), collar protein (gp8) and tail fibers (gp11, gp12 and gp17). The 8 ORFs in the replication/transcription module are exonuclease (ORF20), DNA polymerase (ORF25), DNA primase/helicase (ORF230), DNA endonuclease (ORF32), bacterial RNA polymerase inhibitor (ORF234), DNA ligase (ORF38), DNA-dependent RNA polymerase (ORF42) and RNA polymerase (ORF43). In the host lysis module, one lysin, a holin and Rz-spanin were found. The spanin, holin and lysin proteins were found as (ORF3), (ORF5) and (ORF 31), respectively. No antibiotic resistance gene, phage-coded virulence gene, bacterial virulence gene, or integrase was detected in the genome of phage by Antibiotic Resistance Gene Database and Virulence Factor Database. According to the genomic and bioinformatics analysis, phage LPST153 is safe a potential agent against *Salmonella* infection. The above evidences suggest that phage LPST153 could be used as a potential controlling agent against *Salmonella.*

### 3.5. Comparative Genome Analysis

**Figure 4:**
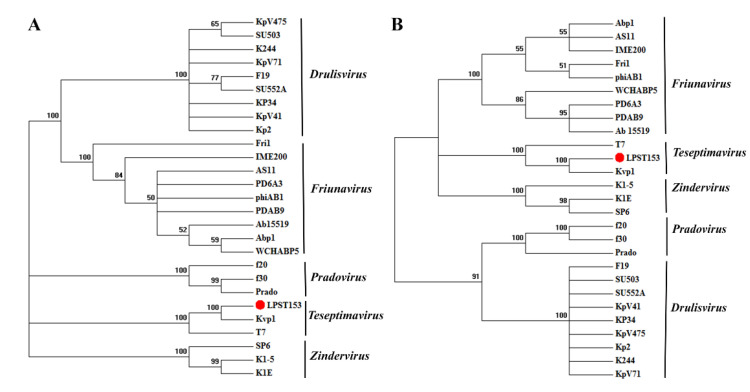
Phylogenetic analysis of phages in different genus of *Autographivirinae* subfamily based on their terminase large subunit. (**A**) and major capsid proteins. (**B**). Phylogenetic trees were constructed using the neighbor-joining method with 1000 bootstrap replications.

When the genome sequence was compared in NCBI database by BlastN analysis, the results showed the genome of phage LPST153 has a high degree of homology with phage BP12A in the database (Genbank Acc. No. KM366096). Phage BP12A is an unknown characteristics phage (there is no related published article yet) and also whether phage has the ability to degrade exopolysaccharides. Other phages offered the average similarity with LPST153 below 90% (Table S2). The phylogenetic trees obtained using terminase large subunit, and major capsid proteins (Figure 4A,B) exhibited similar topologies and supported assignment of phage LPST153 to a sublineage shared by *Kluyvera* phage Kvp1 and *Escherichia* Phage T7, which are the recommended phages of *Teseptimavirus* genus. Phylogenetic analysis showed that LPST153 had high identities with *Teseptimavirus* phage but separated from *Drulisvirus, Friunavirus, Pradovirus* and *Zindervirus* (Figure 4). The genome analysis and phylogenetic results indicated that LPST153 is the new member of the *Teseptimavirus* genus of *Autographivirinae* subfamily (Figure 4 and Table S2).

### 3.6. Application of Phage LPST153 in Controlling Salmonella in Foods and Biofilms

**Figure 5:**
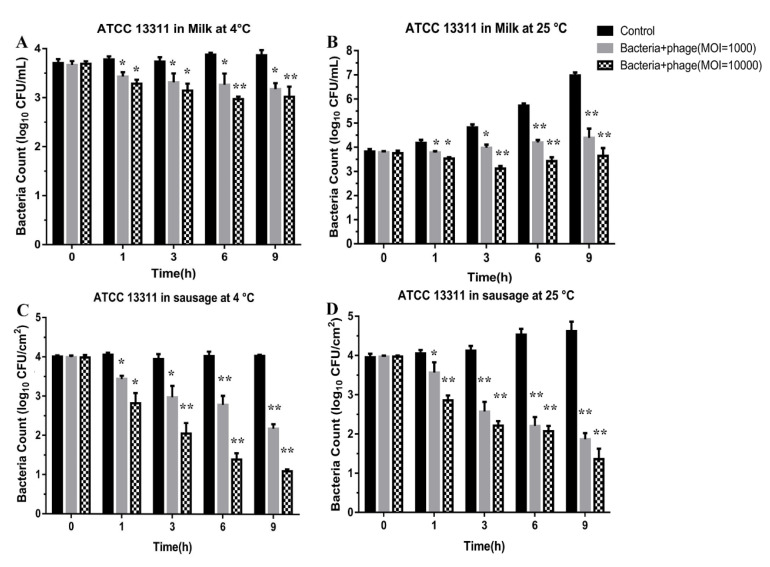
Effectiveness of phage LPST153 in reducing *S.* Typhimurium ATCC 13311 in food matrices. Effect of phage LPST153 on growth of *S*. Typhimurium ATCC 13311 in the milk incubated at (**A**) 4 °C and (**B**) 25 °C and in sausage incubated at (**C**) 4 °C and (**D**) 25 °C. Values represent mean with standard deviation of three determinations. ** Significant at *p* < 0.01; * Significant at *p* < 0.05.

Phage LPST153 has been applied in pasteurized raw milk and raw beef sausage for treatment of artificially contaminated *Salmonella* for evaluating the bactericidal effects of phage in liquid and solid food. In milk with an MOI of 1000, the *Salmonella* viability decreased by 0.7 log CFU/mL (*p* < 0.05) and 2.5 log CFU/mL (*p* < 0.01) at 4 °C and 25 °C after 9 h incubation, respectively (Figure 5A,B). In the case of an MOI of 10,000, a 0.9 log CFU/mL and a 3.3 log CFU/mL (*p* < 0.01) reduction were observed after 9 h incubation at 4 °C and 25 °C, respectively (Figure 5A,B). For sausage samples, LPST153 reduced viable *Salmonella* by 1.8 log CFU/cm^2^ at 4 °C and 2.7 log CFU/cm^2^ (*p* < 0.01) at 25 °C after 12 h incubation with MOI of 1000 and the reduction was separately observed 2.9 log CFU/cm^2^ (*p* < 0.01) with an MOI of 10,000 at 4 °C and 3.3 CFU/cm^2^ at 25 °C after 12 h incubation (Figure 5C,D).

**Figure 6:**
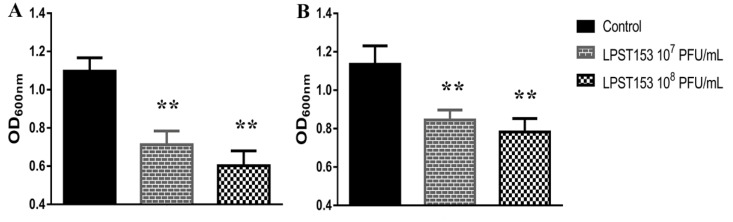
Effect of phage LPST153 on biofilm. (**A**) Effect of phage LPST153 on inhibition of biofilm in 96-well microplate at 30 °C after 72-h incubation, and (**B**) Effect of phage LPST153 on 72-h-old biofilm in 96-well microplate at 30 °C after 12 h postinfection. Values represent mean with standard deviation of six determinations. ** Significant at *p* < 0.01.

Biofilm of *S.* Typhimurium ATCC 13311 was established in 96-well microplates. In the biofilm inhibition assay, phage LPST153 was mixed with *S*. Typhimurium ATCC 13311 before incubation. When 7 log_10_ and 8 log_10_ PFU/mL phage was applied, an approximately 35% and 45% respective inhibition were observed by colorimetric method (Figure 6A). The other method was performed to observe if the LPST153 can reduce the existing biofilm. When 7 log_10_ and 8 log_10_ PFU/mL was applied to the *Salmonella* biofilm, approximately 25% and 31% reduction were observed, respectively (Figure 6B).

## 4. Discussion

Phage LPST153 can form plaques which are surrounded by translucent halos—this is different with all other isolated phages in this study. Since the presence of halos around the clear plaque zone is believed to be a decent indication that the diffusion of the phage enzymatic molecules (such as EPS depolymerases) can be effective in dispersing biofilms [50]. These phages have biotechnological applications in the treatment because it is possible that biofilms were controlled by phage infection and phage has the ability to produce (or to be able to induce) enzymes that can degrade extracellular matrix [52,53]. Hughes et al. have reported that the presence activity of polysaccharide depolymerases from phage SF153b might be the reason to be effective against *Enterobacter agglomerans* biofilms [54]. Furthermore, Siringan et al. have demonstrated that while biofilms are treated with *Campylobacter jejuni* phages (CP8, CP30), this could not only affect the target and lyse cell but also disperse the EPS of the biofilm [55]. Phage LPST153 had capsids possessing icosahedral symmetry and diameter of 51.5 ± 4.5 nm; it also had a short tail (7.5 ± 2.4 nm). Rashid et al. reported that phages belonged to *Podoviridae* family had mean diameter of about 50 to 60 nm and short tail (5 to 10 nm) [56]. It has been confirmed that phage LPST153 belongs to the *Podoviridae* family of the order *Caudovirales*. As suggested by other literatures, phages, which belong to *Podoviridae* family, have the potential application to inhibit biofilm formation [34,50].

Analysis of the lytic and host range showed that phage LPST153 could lyse the four most prevalent *Salmonella* serovars including *S.* Typhimurium, *S.* Enteritidis, *S.* Pullorum and *S.* Gallinarum. Phage LPST153 had active lytic activity against host and could constantly inhibit the growth of host (*S.* Typhimurium ATCC 13311) for up to 12 h at MOIs of 0.1, 1, 10 and 100. In contrast, other phages (FGCSSa1, and PA1307) could inhibit the growth of their host for only 2 to 5 h under MOIs from 2.5 to 1000 [26,57]. Phage LPST153 performed a latent period of 10 min and an average burst size of 113 ± 8 PFU/cell in one-step growth curve results. The latent period and burst size of phage are key factors in considering whether the phage can be selected for biocontrol experiments [58]. It has been proved that high burst size and short latent period are positively related on effectively inactivating bacteria [59]. Resistance to heat and pH are essential for biological control applications. Phage LPST153 can remain high activity in a wide pH range; there is no significant dropping from highly acidic conditions (pH 4) to highly alkaline conditions (pH 12). Phage LPST153 can also survive in the temperature range between 30 and 60 °C for at least 60 min. It was found phage LPST153 was even more tolerant of heat and extreme pH conditions as compared with reports of other *Salmonella* phages. Hoglund et al. reported that the titer of *S.* Typhimurium phage 28B decreased outside the range of pH 5 to 9 or upon exposure to 37 °C [60]. Bao et al. reported that *Salmonella* phage PC2184 was stable over the pH range of 5 to 8 [26]. LPST153 is more likely to remain antibacterial activity under tested conditions, which means it possibly can tolerate similar extreme environments caused by food processing.

The total genome is 39,176 bp and the base composition of 49.1% G+C content is remarkably consistent with other T7-like phages. By comparison, the genomes of T7-like phages range from 37.4 kb (Pseudomonas phage gh-1) to 45.4 kb (Erwinia phage Era103) [61]. No tRNA genes were identified, which was not an unexpected observation since no T7-like phages have been found to harbor them [61]. DNA packaging protein is common for all T7-like phage [62]. The T7 tail complex comprises of a conical tail tube enclosed by six linked tail fibers, which are oligomers of the viral protein gp17 [63]. The six tail fibers were folded against the capsid and positioned equally only after fruitful adsorption to the target host cell surface. Tail fibers binding to the receptor trigger conformational changes resulting in the addition of the extended tail [64].

The first step of a viral infection is the attachment. Six gp17 tail fibers of T7 phage bind to LPS of host bacteria [65]. The short-tailed phage T7 has been shown to reorder its virion proteins while injecting to build a structure that could act as such a DNA ejection conduit, but the details of this structure and functional mechanism remain a mystery [64]. T7 lysozyme hydrolyzes an amide bond in the host cell wall resulting in its release from the cytoplasm. Moreover, T7 lysozyme binds to and inhibits transcription by T7 RNA polymerase. Such inhibition supports the switch to particle assembly [66]. The T7 lysozyme and T7 RNA polymerase complex stimulates phage gene expression during host bacterial infection. The lysozyme binds at a site distant from polymerase active site, signifying an secondary mechanism of inhibition [67]. During lysis process, holins act as gatekeepers which possess an intriguing ability to be triggered at a specific time point, to form big holes in the cytoplasmic membrane of phage-infected bacteria [68]. The lysin function was attributed to ORF31 of LPST153. The lysin of phage LPST153 had the highest similarity with *Kluyvera* phage Kvp1, with a coverage of 100% (similarity, 97%). According to the topology, holins can be grouped into three classes. Class I holins contain more than 95 residues and form three transmembrane domains, class II holins are smaller (65 to 95 residues) and process two transmembrane domains and class III holins contain one transmembrane domain in the central region of the molecule [69]. The holin of LPST153 (ORF5) belongs to class II holin. Type II holins are thought to allow lysin access to the cell wall at the optimal lysis time. After holin and lysin permeabilized the inner membrane and degraded the host peptidoglycans, the spanin complex drives the final step in host lysis by disrupting the outer membrane [70]. Spanin complex disrupts the host outer membrane and participates in cell lysis during virus exit [71].

The potential ability of LPST153 in diverse food matrices was evaluated. In this study, 4 °C (refrigerated temperature) and 25 °C (room temperature) have been applied on raw milk and sausage; they were used as a simulation of liquid and solid food materials in food processing facilities. In the case of milk, the *Salmonella* viability respectively decreased by 2.5 and 3.3 log CFU/mL using the MOI of 1000 and 10,000 at 25 °C. More than 2.5 log reduction of *Salmonella* was observed with MOI of 1000 or 10,000 treatment on sausage samples. Previously reported phages (PA13076, fmb-p and P22) used to reduce *Salmonella* in diverse food base materials [26,38,72]. Phage LPST153 had greater efficiency when compared with phage PA13076 that was reported to reduce S. Enteritidis ATCC 13076 by 1 log_10_ CFU/mL in milk [26]. Wang et al. demonstrated *Salmonella* phage fmb-p1 could decrease *S.* Typhimurium counts by 1.75 log CFU/cm^2^ on duck meat. Goode et al. reported that phage PA13076 led to a reduction in the viability of *Salmonella* with the reduction of 2.0 log CFU/sample in chicken skin. Given the burden associated with the therapeutic management of invasive infections, and particularly with those with biofilm development, phages may play an important role. They may exhibit anti-biofilm properties, even when lytic activity plays no role in planktonic bacteria, despite their dynamic penetration into bacterial biofilms, the higher cellular density and probability of bacterial cells attachment and the expected expression of EPS depolymerase enzymes [73,74]. Many researchers have reported that effective biofilm reduction (1 to 6 log) depends on the biofilm components such as biofilm age, phage effectiveness and treatment time [75,76]. It has been found that treating with a phage LPST15 inhibits (35–45%) and reduces (25–31%) biofilm on 96-well microplate, respectively.

## 5. Conclusions

In this study, LPST153, a novel *Salmonella* lytic phage with halo was isolated and with *S.* Typhimurium ATCC 13311 performing as the host. It has excellent lytic potential against *Salmonella*. LPST153 is effectively able to lyse most prevalent tested serotypes of *Salmonella,* including *S.* Typhimurium, *S.* Enteritidis, *S.* Pullorum and *S.* Gallinarum. Furthermore, the phage has been tested on morphology, lytic range, latent period, burst size, pH stability, and thermal stability. It has been proved that phage LPST153 has potential to reduce *Salmonella* in food matrices and biofilms. After genome sequencing analysis, LPST153 is found to be a new member of the *Teseptimavirus* genus of the *Autographivirinae* subfamily. As a biological control agent, the potential efficiency of LPST153 demonstrates a great ability against *Salmonella* even in complicated food processing environments.

## Figures and Tables

**Table 1:** Host range of phage LPST153.

Bacteria	Source of Strains	Multidrug-Resistant	LPST153/Spot Test	LPST153/EOP
*S.* Typhimurium ATCC 14028	ATCC	N	+	0.18
*S.* Typhimurium ATCC 13311	ATCC	N	+	Host
*S.* Typhimurium LST2 (ST-8)	CI	N	+	0.1
*S.* Typhimurium LST4 (UK-1)	LS	N	+	0.17
*S.* Typhimurium LST6 (LT2)	LS	N	+	1
*S.* Typhimurium SGSC 4903	SGSC	N	+	1
*S.* Typhimurium LST10	LS	Y (CTT, AMP, CZO, GEN, AMK, TOB, CIP, NIT)	+	0.004
*S.* Typhimurium LST11	LS	Y (CTT, AMP, CZO, GEN, AMK, TOB, CIP, SXT, NIT)	+	0.003
*S.* Typhimurium LST14	LS	Y (CZO, CAZ, FEP, TOB, CRO, SXT)	+	0.006
*S.* Typhimurium LST17	LS	Y (AMP, CZO, CAZ, FEP, TOB, CRO, ATM)	+	0.005
*S.* Typhimurium LST18	LS	Y (CTT, AMP, CZO, GEN, AMK, TOB, CIP, SXT, NIT)	+	0.010
*S.* Typhimurium LST19	LS	Y (CTT, AMP, CZO, GEN, AMK, TOB, CRO, CIP, NIT)	+	0.016
*S.* Enteritidis ATCC 13076	ATCC	N	+	0.1
*S.* Enteritidis SJTUF 10978	SJTU	N	+	0.19
*S.* Enteritidis SJTUF 10984	SJTU	N	+	0.17
*S.* Enteritidis SGSC 4901	SGSC	N	+	0.12
*S.* Enteritidis LSE7	LS	Y (CTT, AMP, CZO, GEN, AMK, TOB, CIP, SXT, NIT)	+	0.004
*S.* Enteritidis LSE8	LS	Y (CTT, AMP, CZO, GEN, AMK, TOB, SXT, NIT)	+	0.1
*S.* Enteritidis LSE10	LS	Y (AMP, CZO, NIT)	+	0.15
*S.* Enteritidis LSE15	LS	Y (AMP, CZO, GEN, AMK, TOB, NIT)	+	0.013
*S.* Pullorum LSP1 (CVCC 519)	LS	N	+	0.1
*S.* Pullorum LSP2 (CVCC 534)	LS	N	+	0.1
*S.* Pullorum LSP3	LS	N	+	0.15
*S.* Pullorum LSP4	LS	N	+	0.17
*S.* Pullorum LSP5	LS	N	+	0.13
*S.* Gallinarum LSG1	LS	N	+	0.18
*S.* Gallinarum LSG2	LS	N	+	0.1
*S.* Gallinarum LSG3	LS	N	+	0.1
*S.* Gallinarum LSG4	LS	N	+	0.17
*S.* Gallinarum LSG5	LS	N	+	0.15
*S.* Dublin LSD1 (3710)	LS	N	+	0
*S.* Dublin LSD2 (3723)	LS	N	−	0
*S.* Anatum ATCC 9270	ATCC	N	−	0
*S.* Arizonae CDC 346-86	CDC	N	+	0
*S.* Javiana LSX23 (CVM 35943)	LS	N	+	0.03
*S.* Kentucky LSX24 (CVM 29188)	LS	N	−	0
*S.* Newport E20002725	CDC	N	−	0
*S.* Paratyphi B CMCC 50094	CMCC	N	+	0.19
*S.* Choleraesuls ATCC 10708	ATCC	N	+	0
*S*. Typhi LSX1 (CT18)	LS	N	+	0.1
*S.* Typhi LSX2 (Ty2)	LS	N	+	0.1
*E. coli* LEC1 (F18AC)	TB	N	−	0
*E. coli* LEC2 (C83715)	TB	N	−	0
*E. coli* LEC3 (T10)	TB	N	−	0
*E. coli* LEC4 (DH5α)	TB	N	−	0
*E. coli* LEC6 (BL21)	TB	N	−	0
*A. hydrophila* ZYAH72	LS	N	−	0
*A. hydrophila* ZYAH75	LS	N	−	0
*A. hydrophila* J1	LS	N	−	0
*A. hydrophila* ZYAH91 (D4)	LS	N	−	0
*C. sakazakii* ATCC 12868	ATCC	N	−	0
*C. sakazakii* ATCC 29004	ATCC	N	−	0
*C. sakazakii* ATCC 29544	ATCC	N	−	0
*S. flexneri* CMCC 51572	CMCC	N	−	0
*V. parahaemolyticus* ATCC 17802	ATCC	N	−	0
*V. parahaemolyticus* ATCC 33846	ATCC	N	−	0
*P. aeruginosa* ATCC 7853	ATCC	N	−	0
*S. aureus* ATCC 6538	ATCC	N	−	0
*S. aureus* ATCC 8095	ATCC	N	−	0
*S. aureus* ATCC 29213	ATCC	N	−	0
*Listeria* ATCC 19114	ATCC	N	−	0
*Listeria* ATCC 19115	ATCC	N	−	0
*Streptococcus suis* LSM122 (P1/7)	LS	N	−	0
*Streptococcus suis* LSM123 (SC19)	LS	N	−	0
*L. acidophilus* ATCC SD5221	ATCC	N	−	0

Abbreviation: ATCC, American Type Culture Collection; CI, Clinical isolate; SGSC, *Salmonella* Genetic Stock Center; LS, Lab Stock; SJTU, Shanghai Jiao Tong University; CDC, Centers for Disease Control and Prevention; TB, TransGen Biotech; CMCC, National Center for Medical Culture Collection; Y, Yes; N, No; CTT, cefotetan; AMP, ampicillin; CZO, cefazolin; CAZ, ceftazidime; FEP, cefepime; GEN, gentamicin; AMK, amikacin; TOB, tobramycin; CRO, ceftriaxone, ATM, aztreonam; CIP, ciprofloxacin; SXT, pediatric compound sulfamethoxazole tablets; NIT, nitrofurantoin. EOP 0.5 to 1.0, high efficiency; EOP 0.2 to <0.5, moderate efficiency; 0.001 to <0.2, low efficiency; and <0.001, inefficient.

## Supplementary Materials

The following are available online at https://www.mdpi.com/2076-2607/8/7/1089/s1. Table S1. Functional annotation of LPST153 CDSs. Table S2. Comparison of phages against the LPST153 genome.
