# Supplementary material for: Characterization of Salmonella Phage LPST153 That Effectively Targets Most Prevalent Salmonella Serovars
Source: Microorganisms. 2020 Jul 21;8(7):1089. doi: 10.3390/microorganisms8071089 (PMC7409278; doi:10.3390/microorganisms8071089)
Supplement: Supplementary file 1 [file microorganisms-08-01089-s001.pdf]

microorganisms

MDPI

Supplementary Materials

Characterization of *Salmonella* Phage LPST153 That Effectively Target Most Prevalent *Salmonella* Serovars

Md. Sharifull Islam[1,2,3], Yang Hu[4], Md. Furkanur Rahaman Mizan[5], Ting Yan[3], Ishatur Nime[3], Yang Zhou[1,2,*] and Jinquan Li[2,3,6]

[1] College of Fisheries, Huazhong Agricultural University, Wuhan, Hubei, People's Republic of China; zhouyang@mail.hzau.edu.cn (Y.Z)
[2] State Key Laboratory of Agricultural Microbiology, Huazhong Agricultural University, Wuhan, Hubei, People's Republic of China; smbgb101287@yahoo.com (M.S.I.); zhouyang@mail.hzau.edu.cn (Y.Z); lijinquan2007@gmail.com (J. Li)
[3] Key Laboratory of Environment Correlative Dietology, College of Food Science and Technology, Huazhong Agricultural University, Wuhan, Hubei, People's Republic of China; smbgb101287@yahoo.com (M.S.I.); yantingau@163.com (T.Y.); smbgb101287@gmail.com (I.N.); lijinquan2007@gmail.com (J. Li.)
[4] Division of Microbiology, Brewing and Biotechnology, School of Biosciences, University of Nottingham, S utton Bonington Campus LE12 5RD, UK; stxyh26@exmail.nottingham.ac.uk (Y.H.)
[5] Department of Food Science and Technology, Chung-Ang University, Anseong, Gyunggido, Republic of Korea; rahaman4162@cau.ac.kr
[6] Laboratory of Bacterial Pathogenesis and Immunology, The Rockefeller University, New York, New York, USA; lijinquan2007@gmail.com (J. Li.)
* Correspondence: zhouyang@mail.hzau.edu.cn (Y.Z)

Received: date; Accepted: date; Published: date

Table S1. Functional annotation of LPST153 CDSs

ORF	Gene Positions	Functions	Name	
1	410-565	gp19.5 [Enterobacteria phage BA14]	PHA00406	PHA00406
2	834-2591	DNA packaging protein B [Erwinia phage FE44]		
3	2601-3032	endopeptidase Rz [Salmonella phage BP12A]		
4	3131-3397	DNA packaging protein A [Salmonella phage BP12A]	HNH_3	pfam13392
5	3390-3596	class II holing [Salmonella phage BP12A]		
6	3609-5549	tail fibers protein [Salmonella phage BP12A]		
7	5621-9568	gp16 [Enterobacteria phage 285P]	PhageP22-tail super family	cl07762
8	9592-11868	gp15 [Enterobacteria phage 285P]	RPA like super family	cl09930
9	11871-12485	gp14 [Enterobacteria phage BA14]	ERF	pfam04404
10	12488-12904	gp13 [Yersinia phage Yepe2]	PHA00432	PHA00432
11	12984-15359	tail tubular protein B [Salmonella phage BP12A]		
12	15395-15985	tail tubular protein A [Salmonella phage BP12A]	PHA00428	PHA00428
13	16193-17227	major capsid protein [Salmonella phage BP12A]	PHA00201	PHA00201
14	17395-18306	capsid and scaffold protein [Escherichia phage P694]	PHA00435	PHA00435
15	18378-19979	collar protein [Escherichia phage Ebrios]	Head-tail_con	pfam12236
16	19992-20330	tail assembly protein [Yersinia phage vB_YenP_AP10]	PHA00437	PHA00437
17	20348-20623	gp6.7 [Enterobacteria phage 285P]	PHA00441	PHA00441
18	20643-20888	gp6.5 [Enterobacteria phage 285P]	PHA00438	PHA00438
19	20839-20931	unknown product [Enterobacteria phage BA14]		
20	21056-21970	exonuclease [Escherichia phage vB_EcoP_S523]	PHA00439	PHA00439
21	21967-22254	gp5.8 [Erwinia phage vB_EamP-L1]		
22	22251-22460	HNS binding protein [Escherichia phage P483]	PHA00422	PHA00422
23	22460-22753	gp5.5 [Enterobacteria phage BA14]	DUF2675 super family	cl26474
24	22764-22991	hypothetical protein BP12A_20A [Salmonella phage BP12A]		
25	22991-25105	DNA polymerase [Salmonella phage BP12A]	DNA_pol_A super family	cl02626
26	25118-25534	gp4.7 [Enterobacteria phage BA14]	GH3 super family	cl21606

27	25622-25924	gp4.5 [Enterobacteria phage BA14]	PHA00446	PHA00446
28	25938-26150	gp4.3 [Enterobacteria phage BA14]	PHA00448	PHA00448
29	26143-26337	gp4.2 [Yersinia phage Yep-phi]	PHA00476 super family	cl10223
30	26282-27994	DNA primase/helicase [Salmonella phage BP12A]	GP4d_helicase	cd01122
31	28163-28618	lysozyme/N-acetylmuramoyl-L-alanine amidase[Klebsiella phage vB_KpnP_PRA33]S	PHA00447	PHA00447
32	28615-29061	endonuclease [Escherichia phage P483]	PHA00159	PHA00159
33	29061-29768	DNA-binding protein [Salmonella phage BP12A]	PHA00458	PHA00458
34	29836-30027	bacterial RNA polymerase inhibitor [Kluyvera phage Kvp1]	PHA00457	PHA00457
35	30024-30122	gp1.8 [Enterobacteria phage BA14]	PHA00456	PHA00456
36	30115-30465	gp1.7 [Yersinia phage Yep-phi]	DUF3310	pfam11753
37	30465-30725	gp1.6 [Enterobacteria phage BA14]	PHA00455	PHA00455
38	30905-31978	DNA ligase [Escherichia phage P694]	PHA00454	PHA00454
39	32079-32339	deoxyguanosine inhibitor [Pectobacterium phage PP74]	DUF2745 super family	cl26487
40	32341-32490	gp1.1 [Enterobacteria phage BA14]	PHA01327	PHA01327
41	32584-33141	gp1.1 [Enterobacteria phage BA14]		
42	33230-34237	DNA-dependent RNA polymerase [Salmonella phage BP12A]	RNA_pol super family	cl25973
43	34227-36008	RNA polymerase [Escherichia phage vB_EcoP_S523]	PHA00452	PHA00452
44	36082-37185	protein kinase [Salmonella phage BP12A]	PHA00451	PHA00451
45	37199-37417	0.6B protein [Enterobacteria phage T7]		
46	37401-37598	gp0.6A [Enterobacteria phage BA14]		
47	37598-37741	hypothetical protein [Yersinia phage phiYe-F10]		
48	37744-38202	S-adenosyl-L-methionine hydrolase [Escherichia phage S523]		

Table S2. Comparison of phages against the LPST153 genome.

Phage	Country	Accession	Genome length(bp)	E value	BLASTN score	Query cover	Identify	Genus	Ref.
Salmonella phage BP12A	Canada	KM366096	39696	0	57061	95%	972%	*Teseptimavirus*	unpublished
Enterobacteria phage BA14	USA	EU734171	39816	0	42391	89%	85.45%	*Teseptimavirus*	unpublished
Enterobacteria phage 285P	China	GQ468526	39270	0	42134	88%	85.43%	*Teseptimavirus*	unpublished
Escherichia phage S523	China	MH031343	39825	0	42664	89%	89.67%	*Teseptimavirus*	unpublished
Salmonella phage BSP161	Korea	MG471392	39688	0	42454	88%	87.69%	*Teseptimavirus*	unpublished
Escherichia phage P694	China	KP090454	40477	0	41407	85%	87.38%	*Teseptimavirus*	Chen et al 2016
Kluyvera phage Kvp1	Canada	FJ194439	39472	0	42708	87%	878%	*Teseptimavirus*	Lingohr at al 2008
Escherichia phage D226	China	MN080711	40050	0	43340	88%	86.86%	*Teseptimavirus*	unpublished
Erwinia phage FE44	Ukraine	KF700371	39860	0	42714	88%	89.24%	*Teseptimavirus*	Chen et al 2016
Yersinia phage Yep-phi	China	HQ333270	38616	0	34941	83%	85.74%	*Teseptimavirus*	Zhao et al 2011
Yersinia phage Yepe2	USA	EU734170	38677	0	34763	82%	85.68%	*Teseptimavirus*	unpublished
Yersinia phage YpP-G	USA	JQ965702	39415	0	34763	82%	85.65%	*Teseptimavirus*	Rashid Et,al, 2012
Shigella phageVB_Ship_A7	China	MK685668	40058	0	38491	82%	89.76%	*Teseptimavirus*	unpublished
Escherichia phage P483	China	NC_028822	40829	0	41663	89%	85.63%	*Teseptimavirus*	Chen et al 2016
Pectobacterium phage PP74	Russian	KY084243	39790	0	37527	84%	85.96%	*Teseptimavirus*	Bugaeva at al 2018
